# Supplementary material for: A Likelihood Approach for Real-Time Calibration of Stochastic Compartmental Epidemic Models
Source: PLoS Comput Biol. 2017 Jan 17;13(1):e1005257. doi: 10.1371/journal.pcbi.1005257 (PMC5240920; doi:10.1371/journal.pcbi.1005257)
Supplement: S1 File — (TAR.GZ) [file pcbi.1005257.s014.tar.gz › HSPH_Online-SI-Revision/MSS10/n100-mild/n100-mild_NEW2_table8.pdf]

1	newly 3 weeks spec	114.	106.11	{41., 163.}	31.2193	0.173979	newly 3 weeks cumul	602.	535.7	{281., 710.}	23.1096	0.123676	newly 5 weeks spec	30.	42.74	{8., 81.}	80.2667	0.336861	newly 5 weeks cumul	708.	644.71	{316., 887.}	24.9195	0.132348	newly 44 weeks cumul	814.	717.42	{360., 1028.}	26.3243	0.143567
2	newly 3 weeks spec	204.	198.32	{145., 247.}	16.0588	0.0730168	newly 3 weeks cumul	827.	923.56	{751., 1072.}	15.5695	0.0623112	newly 5 weeks spec	99.	88.34	{55., 129.}	24.0606	0.121232	newly 5 weeks cumul	1036.	1143.11	{916., 1341.}	15.1419	0.0609949	newly 43 weeks cumul	1187.	1309.65	{1079., 1589.}	15.6268	0.0623551
3	newly 3 weeks spec	108.	107.05	{58., 162.}	29.0833	0.134246	newly 3 weeks cumul	712.	545.25	{340., 728.}	26.0154	0.142639	newly 5 weeks spec	44.	44.32	{14., 75.}	45.4545	0.248365	newly 5 weeks cumul	820.	658.65	{405., 895.}	24.7329	0.133032	newly 46 weeks cumul	845.	729.53	{476., 976.}	22.7917	0.117765
4	newly 3 weeks spec	19.	23.07	{6., 41.}	60.2632	0.284659	newly 3 weeks cumul	149.	149.37	{80., 217.}	29.8456	0.137348	newly 5 weeks spec	0.1	6.38	{0., 18.}	6338.	1.24778	newly 5 weeks cumul	155.	168.51	{87., 253.}	35.3742	0.151764	newly 50 weeks cumul	155.	175.74	{87., 267.}	39.3806	0.161303
5	newly 3 weeks spec	147.	168.14	{116., 216.}	25.6054	0.102138	newly 3 weeks cumul	785.	804.77	{641., 962.}	14.3605	0.0626411	newly 5 weeks spec	78.	66.84	{26., 107.}	34.8462	0.193323	newly 5 weeks cumul	977.	980.3	{741., 1219.}	15.3163	0.0684849	newly 46 weeks cumul	1144.	1098.18	{840., 1400.}	15.6469	0.0735036
6	newly 3 weeks spec	116.	135.33	{85., 184.}	28.5603	0.109916	newly 3 weeks cumul	649.	676.36	{506., 837.}	15.5069	0.0646631	newly 5 weeks spec	30.	58.61	{31., 95.}	100.5	0.277924	newly 5 weeks cumul	747.	826.44	{610., 1063.}	18.8648	0.0744405	newly 44 weeks cumul	826.	928.66	{642., 1243.}	21.6925	0.0847013
7	newly 3 weeks spec	14.	15.25	{0., 41.}	106.071	0.91216	newly 3 weeks cumul	126.	102.55	{10., 236.}	58.9762	0.42827	newly 5 weeks spec	0.1	3.57	{0., 11.}	3612.	0.566631	newly 5 weeks cumul	128.	114.46	{10., 275.}	64.9531	0.441638	newly 49 weeks cumul	128.	117.55	{10., 279.}	66.8047	0.44541
8	newly 3 weeks spec	39.	59.82	{20., 110.}	75.7436	0.292219	newly 3 weeks cumul	302.	319.66	{151., 492.}	34.4503	0.179234	newly 5 weeks spec	15.	24.56	{2., 57.}	110.267	0.483804	newly 5 weeks cumul	333.	384.94	{194., 602.}	40.5766	0.191162	newly 43 weeks cumul	361.	429.89	{194., 702.}	45.7091	0.20786
9	newly 3 weeks spec	85.	88.89	{58., 122.}	24.8588	0.105615	newly 3 weeks cumul	398.	461.78	{352., 565.}	21.	0.0805852	newly 5 weeks spec	41.	31.75	{12., 53.}	39.439	0.231422	newly 5 weeks cumul	509.	546.57	{411., 676.}	17.4558	0.0711948	newly 48 weeks cumul	521.	596.47	{440., 772.}	22.4818	0.086546
10	newly 3 weeks spec	178.	146.51	{96., 207.}	25.5562	0.133875	newly 3 weeks cumul	763.	687.05	{515., 907.}	18.6304	0.0906322	newly 5 weeks spec	52.	61.82	{26., 93.}	42.1538	0.17389	newly 5 weeks cumul	913.	842.73	{624., 1125.}	19.0372	0.0909011	newly 42 weeks cumul	1004.	967.72	{658., 1307.}	19.1036	0.0895108
11	newly 3 weeks spec	68.	80.36	{40., 125.}	39.	0.160861	newly 3 weeks cumul	409.	461.79	{305., 606.}	25.2543	0.102533	newly 5 weeks spec	9.	26.81	{8., 53.}	209.222	0.461606	newly 5 weeks cumul	430.	535.33	{339., 725.}	33.6535	0.126173	newly 46 weeks cumul	447.	572.24	{346., 776.}	36.953	0.135882
12	newly 3 weeks spec	387.	285.69	{230., 340.}	26.1783	0.13616	newly 3 weeks cumul	1436.	1258.15	{1114., 1402.}	12.8558	0.0612263	newly 5 weeks spec	224.	135.81	{103., 169.}	39.3705	0.225442	newly 5 weeks cumul	1967.	1592.42	{1404., 1806.}	19.0595	0.0939058	newly 42 weeks cumul	2777.	1905.51	{1645., 2195.}	31.3824	0.166202
13	newly 3 weeks spec	44.	69.33	{22., 117.}	76.7045	0.249933	newly 3 weeks cumul	425.	362.49	{195., 542.}	28.2	0.153052	newly 5 weeks spec	11.	22.58	{1., 45.}	145.091	0.502407	newly 5 weeks cumul	440.	426.23	{229., 640.}	29.0705	0.142415	newly 47 weeks cumul	442.	458.1	{230., 666.}	32.1267	0.148593
14	newly 3 weeks spec	73.	36.23	{11., 67.}	52.7808	0.41469	newly 3 weeks cumul	262.	218.08	{113., 316.}	28.	0.157757	newly 5 weeks spec	18.	10.95	{1., 26.}	60.7222	0.581897	newly 5 weeks cumul	346.	250.34	{126., 367.}	32.6879	0.199262	newly 49 weeks cumul	354.	260.36	{127., 394.}	32.565	0.198153
15	newly 3 weeks spec	78.	49.35	{20., 78.}	39.9103	0.289201	newly 3 weeks cumul	442.	304.22	{176., 450.}	33.2172	0.206777	newly 5 weeks spec	22.	14.85	{1., 32.}	56.6818	0.506531	newly 5 weeks cumul	504.	345.2	{201., 509.}	33.7222	0.212657	newly 46 weeks cumul	595.	360.99	{202., 515.}	40.3782	0.263204
16	newly 3 weeks spec	250.	225.12	{146., 317.}	24.008	0.118328	newly 3 weeks cumul	1232.	1087.2	{788., 1330.}	17.3961	0.0865063	newly 5 weeks spec	117.	94.47	{44., 150.}	33.2051	0.197217	newly 5 weeks cumul	1525.	1325.01	{953., 1676.}	18.7443	0.0947136	newly 42 weeks cumul	1724.	1488.51	{978., 1899.}	19.5702	0.100726
17	newly 3 weeks spec	47.	53.33	{25., 82.}	42.617	0.181474	newly 3 weeks cumul	382.	326.43	{181., 469.}	25.7618	0.136004	newly 5 weeks spec	36.	15.8	{3., 33.}	59.3333	0.550529	newly 5 weeks cumul	445.	370.55	{209., 525.}	26.7438	0.142635	newly 48 weeks cumul	515.	388.11	{220., 545.}	30.2427	0.17073
18	newly 3 weeks spec	0.1	3.46	{0., 12.}	3484.	0.670966	newly 3 weeks cumul	28.	31.86	{2., 64.}	73.2857	0.399739	newly 5 weeks spec	0.1	0.68	{0., 0.}	764.	0.132183	newly 5 weeks cumul	28.	34.13	{2., 69.}	81.0357	0.41016	newly 52 weeks cumul	28.	34.39	{2., 69.}	81.9643	0.410692
19	newly 3 weeks spec	126.	102.13	{51., 150.}	28.6587	0.159946	newly 3 weeks cumul	683.	540.52	{343., 719.}	23.3968	0.127767	newly 5 weeks spec	78.	39.34	{8., 67.}	51.1538	0.41246	newly 5 weeks cumul	871.	642.31	{391., 861.}	27.5396	0.15527	newly 43 weeks cumul	985.	698.33	{400., 918.}	30.5817	0.175541
20	newly 3 weeks spec	0.1	21.18	{0., 49.}	21112.	1.87952	newly 3 weeks cumul	49.	158.77	{53., 275.}	232.306	0.479572	newly 5 weeks spec	0.1	5.46	{0., 18.}	5480.	0.773624	newly 5 weeks cumul	49.	175.45	{53., 319.}	265.735	0.508797	newly 52 weeks cumul	49.	178.47	{53., 334.}	271.898	0.512712
21	newly 3 weeks spec	25.	10.57	{0., 25.}	70.44	1.01895	newly 3 weeks cumul	110.	95.23	{14., 173.}	45.1	0.298887	newly 5 weeks spec	10.	1.67	{0., 6.}	89.9	1.63882	newly 5 weeks cumul	146.	100.5	{14., 178.}	45.3699	0.349352	newly 52 weeks cumul	154.	101.51	{14., 182.}	45.9416	0.361733
22	newly 3 weeks spec	390.	364.03	{303., 424.}	11.1513	0.0518157	newly 3 weeks cumul	1699.	1570.78	{1389., 1753.}	9.08652	0.0421659	newly 5 weeks spec	240.	189.48	{142., 234.}	23.1917	0.119133	newly 5 weeks cumul	2286.	2022.37	{1774., 2280.}	12.2209	0.0580271	newly 42 weeks cumul	2969.	2483.14	{2142., 2818.}	16.6265	0.0810552
23	newly 3 weeks spec	51.	42.85	{16., 63.}	32.9216	0.191388	newly 3 weeks cumul	271.	258.96	{137., 362.}	26.1255	0.130483	newly 5 weeks spec	0.1	16.39	{1., 38.}	16306.	1.92223	newly 5 weeks cumul	288.	301.65	{145., 431.}	30.7257	0.141133	newly 47 weeks cumul	288.	323.32	{150., 462.}	34.5069	0.148784
24	newly 3 weeks spec	1.	4.53	{0., 15.}	453.	0.904156	newly 3 weeks cumul	56.	46.67	{7., 93.}	54.8393	0.364502	newly 5 weeks spec	0.1	0.87	{0., 1.}	948.	0.190916	newly 5 weeks cumul	56.	49.92	{7., 101.}	60.25	0.376852	newly 51 weeks cumul	56.	50.46	{7., 101.}	61.2143	0.378203
25	newly 3 weeks spec	333.	243.02	{191., 295.}	27.6276	0.146807	newly 3 weeks cumul	1455.	1147.84	{970., 1349.}	21.1656	0.10675	newly 5 weeks spec	128.	112.37	{70., 158.}	23.4297	0.116822	newly 5 weeks cumul	1803.	1423.02	{1222., 1695.}	21.4443	0.10853	newly 42 weeks cumul	2085.	1642.08	{1305., 1954.}	21.8849	0.111924
26	newly 3 weeks spec	134.	127.22	{77., 173.}	21.9552	0.104937	newly 3 weeks cumul	702.	633.39	{495., 768.}	14.3917	0.0693759	newly 5 weeks spec	48.	53.18	{22., 87.}	40.375	0.186114	newly 5 weeks cumul	832.	770.03	{574., 964.}	15.2608	0.072644	newly 44 weeks cumul	1092.	865.87	{626., 1104.}	22.9789	0.12094
27	newly 3 weeks spec	208.	196.65	{143., 247.}	16.2163	0.0759151	newly 3 weeks cumul	1021.	924.83	{758., 1081.}	12.8119	0.0611936	newly 5 weeks spec	93.	82.55	{53., 110.}	22.0108	0.110557	newly 5 weeks cumul	1280.	1134.39	{929., 1340.}	14.0586	0.0680563	newly 42 weeks cumul	1520.	1292.66	{1031., 1561.}	16.6513	0.0826393
28	newly 3 weeks spec	419.	272.86	{197., 352.}	34.8783	0.195868	newly 3 weeks cumul	1620.	1232.23	{966., 1473.}	24.0389	0.124549	newly 5 weeks spec	296.	123.44	{76., 171.}	58.2973	0.405888	newly 5 weeks cumul	2248.	1541.14	{1197., 1857.}	31.444	0.170224	newly 43 weeks cumul	3114.	1811.	{1362., 2204.}	41.8433	0.242788
29	newly 3 weeks spec	193.	155.22	{57., 237.}	32.3109	0.207562	newly 3 weeks cumul	755.	697.75	{405., 960.}	24.0623	0.125457	newly 5 weeks spec	61.	67.84	{10., 135.}	59.4426	0.397042	newly 5 weeks cumul	915.	871.39	{486., 1251.}	26.8995	0.136864	newly 45 weeks cumul	1584.	1002.6	{486., 1455.}	38.4444	0.24549
30	newly 3 weeks spec	34.	32.15	{8., 55.}	46.6765	0.305913	newly 3 weeks cumul	205.	196.65	{90., 308.}	31.0683	0.155121	newly 5 weeks spec	6.	8.17	{0., 22.}	107.5	0.668304	newly 5 weeks cumul	220.	221.06	{97., 337.}	32.5273	0.155526	newly 47 weeks cumul	225.	232.07	{103., 368.}	35.0711	0.163644
31	newly 3 weeks spec	208.	159.46	{93., 221.}	28.2404	0.159023	newly 3 weeks cumul	1042.	799.75	{569., 1032.}	24.8762	0.13288	newly 5 weeks spec	80.	64.65	{21., 109.}	38.9375	0.229039	newly 5 weeks cumul	1231.	964.41	{640., 1262.}	24.5321	0.130859	newly 44 weeks cumul	1394.	1061.93	{685., 1449.}	27.0423	0.147271
32	newly 3 weeks spec	553.	352.79	{292., 407.}	36.2043	0.199016	newly 3 weeks cumul	1991.	1518.05	{1344., 1681.}	23.7544	0.119212	newly 5 weeks spec	288.	183.57	{142., 222.}	36.2743	0.204317	newly 5 weeks cumul	2687.	1956.26	{1682., 2201.}	27.1954	0.139775	newly 42 weeks cumul	3562.	2425.44	{1976., 2809.}	31.9079	0.169757
33	newly 3 weeks spec	0.1	10.33	{0., 43.}	10344.	0.89904	newly 3 weeks cumul	6.	114.13	{17., 250.}	1805.83	1.11189	newly 5 weeks spec	0.1	1.85	{0., 0.}	1930.	0.209009	newly 5 weeks cumul	6.	119.95	{17., 270.}	1902.83	1.1248	newly 52 weeks cumul	6.	125.07	{17., 294.}	1988.17	1.13012
34	newly 3 weeks spec	24.	12.31	{0., 27.}	58.625	0.717785	newly 3 weeks cumul	66.	81.38	{20., 135.}	59.7576	0.272909	newly 5 weeks spec	13.	2.39	{0., 10.}	82.6923	1.4951	newly 5 weeks cumul	105.	89.37	{20., 155.}	42.9619	0.285375	newly 48 weeks cumul	110.	91.91	{20., 176.}	43.9909	0.2957
35	newly 3 weeks spec	0.1	1.47	{0., 0.}	1550.	0.192882	newly 3 weeks cumul	0.1	24.76	{4., 54.}	24660.	2.18693	newly 5 weeks spec	0.1	0.	{0., 0.}	100.	0.	newly 5 weeks cumul	0.1	24.88	{4., 54.}	24780.	2.18729	newly 52 weeks cumul	0.1	24.88	{4., 54.}	24780.	2.18729
36	newly 3 weeks spec	107.	26.35	{0., 64.}	75.3925	1.10519	newly 3 weeks cumul	314.	177.8	{42., 339.}	49.5732	0.393291	newly 5 weeks spec	47.	5.34	{0., 22.}	88.766	2.03511	newly 5 weeks cumul	444.	195.32	{42., 374.}	57.4505	0.496045	newly 52 weeks cumul	542.	198.48	{42., 374.}	63.4945	0.57303
37	newly 3 weeks spec	66.	64.44	{33., 96.}	32.1212	0.15504	newly 3 weeks cumul	452.	372.16	{230., 493.}	22.7168	0.122132	newly 5 weeks spec	38.	22.57	{7., 41.}	49.2895	0.360473	newly 5 weeks cumul	556.	433.94	{286., 579.}	25.1835	0.138373	newly 45 weeks cumul	581.	460.46	{300., 610.}	24.7883	0.135519
38	newly 3 weeks spec	0.1	1.64	{0., 4.}	1704.	0.304909	newly 3 weeks cumul	6.	19.36	{4., 40.}	239.	0.446983	newly 5 weeks spec	0.1	0.4	{0., 0.}	496.	0.0388081	newly 5 weeks cumul	6.	20.72	{4., 41.}	261.667	0.454524	newly 52 weeks cumul	6.	20.92	{4., 41.}	265.	0.455104
39	newly 3 weeks spec	62.	23.88	{3., 50.}	63.0323	0.683017	newly 3 weeks cumul	248.	154.8	{46., 248.}	43.	0.311824	newly 5 weeks spec	8.	5.81	{0., 19.}	90.875	1.07988	newly 5 weeks cumul	278.	172.44	{49., 285.}	43.5612	0.32144	newly 49 weeks cumul	290.	180.83	{49., 314.}	44.5759	0.330462
40	newly 3 weeks spec	1.	7.28	{0., 22.}	700.	0.915302	newly 3 weeks cumul	22.	65.59	{14., 122.}	215.318	0.461229	newly 5 weeks spec	0.1	1.03	{0., 2.}	1098.	0.253879	newly 5 weeks cumul	22.	68.8	{14., 126.}	229.636	0.473361	newly 51 weeks cumul	22.	69.55	{14., 132.}	233.045	0.476579
41	newly 3 weeks spec	9.	17.51	{1., 38.}	137.444	0.493673	newly 3 weeks cumul	150.	118.87	{53., 201.}	38.6333	0.232803	newly 5 weeks spec	0.1	4.25	{0., 14.}	4260.	0.806165	newly 5 weeks cumul	158.	131.16	{60., 230.}	40.5443	0.237625	newly 52 weeks cumul	158.	135.11	{60., 252.}	41.6899	0.238571
42	newly 3 weeks spec	40.	12.29	{0., 31.}	70.975	1.01408	newly 3 weeks cumul	178.	120.12	{32., 214.}	46.0449	0.341265	newly 5 weeks spec	2.	2.09	{0., 5.}	160.5	1.08472	newly 5 weeks cumul	187.	128.45	{32., 237.}	47.3636	0.349382	newly 49 weeks cumul	187.	129.51	{32., 237.}	47.7273	0.35014
43	newly 3 weeks spec	34.	24.94	{4., 49.}	49.9412	0.408412	newly 3 weeks cumul	177.	154.29	{55., 246.}	37.1695	0.212624	newly 5 weeks spec	7.	7.84	{0., 24.}	114.	0.914074	newly 5 weeks cumul	204.	176.28	{60., 273.}	38.3431	0.221942	newly 52 weeks cumul	209.	182.58	{60., 283.}	39.2632	0.225532
44	newly 3 weeks spec	5.	17.2	{3., 35.}	262.	0.519551	newly 3 weeks cumul	94.	120.02	{47., 207.}	52.4894	0.218769	newly 5 weeks spec	0.1	4.05	{0., 13.}	4048.	0.857358	newly 5 weeks cumul	94.	132.23	{57., 235.}	60.7128	0.230675	newly 51 weeks cumul	94.	136.04	{57., 236.}	64.1915	0.237795
45	newly 3 weeks spec	93.	122.78	{77., 164.}	38.9032	0.139007	newly 3 weeks cumul	558.	632.04	{468., 769.}	20.6559	0.0816769	newly 5 weeks spec	65.	46.85	{21., 74.}	35.4	0.214806	newly 5 weeks cumul	698.	752.31	{540., 936.}	19.159	0.0794767	newly 47 weeks cumul	726.	827.95	{596., 1074.}	23.1033	0.0912533
46	newly 3 weeks spec	37.	63.75	{29., 97.}	80.5676	0.242889	newly 3 weeks cumul	384.	424.03	{284., 562.}	25.3099	0.103825	newly 5 weeks spec	4.	18.23	{2., 36.}	372.75	0.651914	newly 5 weeks cumul	404.	476.43	{314., 643.}	30.245	0.117475	newly 48 weeks cumul	409.	495.96	{318., 667.}	32.6553	0.124382
47	newly 3 weeks spec	33.	26.5	{7., 49.}	44.6061	0.323371	newly 3 weeks cumul	146.	176.53	{82., 254.}	40.1849	0.161469	newly 5 weeks spec	17.	6.96	{0., 15.}	71.5294	1.00128	newly 5 weeks cumul	191.	197.47	{89., 296.}	33.8482	0.159296	newly 48 weeks cumul	201.	205.65	{89., 317.}	35.0199	0.16622
48	newly 3 weeks spec	101.	81.22	{41., 122.}	31.2079	0.178023	newly 3 weeks cumul	455.	450.02	{289., 587.}	21.3934	0.0993328	newly 5 weeks spec	16.	27.28	{6., 55.}	101.375	0.317844	newly 5 weeks cumul	527.	523.94	{340., 699.}	22.9715	0.106126	newly 43 weeks cumul	536.	560.31	{351., 787.}	26.1026	0.114729
49	newly 3 weeks spec	21.	27.26	{0., 68.}	106.857	0.843624	newly 3 weeks cumul	128.	164.41	{31., 326.}	74.5703	0.337671	newly 5 weeks spec	0.1	5.92	{0., 21.}	5940.	0.772083	newly 5 weeks cumul	131.	183.94	{33., 365.}	84.458	0.356321	newly 49 weeks cumul	131.	190.29	{33., 384.}	89.3053	0.367155
50	newly 3 weeks spec	8.	9.68	{0., 30.}	124.	1.0357	newly 3 weeks cumul	96.	91.71	{23., 183.}	49.9896	0.292686	newly 5 weeks spec	0.1	1.87	{0., 5.}	1932.	0.330518	newly 5 weeks cumul	102.	97.62	{23., 196.}	52.0588	0.304126	newly 50 weeks cumul	102.	99.73	{23., 203.}	53.9118	0.308224
